# Significance of Metabolite Ratios in the Interpretation of Segmental Hair Testing Results—Differentiation of Single from Chronic Morphine Use in a Case Series

**DOI:** 10.3390/metabo11080557

**Published:** 2021-08-22

**Authors:** Milena M. Madry, Sandra N. Poetzsch, Andrea E. Steuer, Thomas Kraemer, Markus R. Baumgartner

**Affiliations:** 1 Center for Forensic Hair Analytics, Zurich Institute of Forensic Medicine, University of Zurich, 8006 Zurich, Switzerland; markus.baumgartner@irm.uzh.ch; 2 Department of Forensic Pharmacology & Toxicology, Zurich Institute of Forensic Medicine, University of Zurich, 8057 Zurich, Switzerland; sandra.poetzsch@irm.uzh.ch (S.N.P.); andrea.steuer@irm.uzh.ch (A.E.S.); thomas.kraemer@irm.uzh.ch (T.K.)

**Keywords:** segmental hair analysis, morphine, hydromorphone, metabolite ratios, intoxication, hair wash solution, hair results interpretation, dose–hair concentration correlation

## Abstract

In morphine intoxication cases, forensic toxicologists are frequently confronted with the question of if the individual was opioid-tolerant or opioid-naïve, which can be investigated by hair analysis. However, interpretation of results can be challenging. Here, we report on hair testing for morphine and its metabolite hydromorphone following morphine intoxication without tolerance and upon chronic use. Two consecutive hair samples were collected after a non-fatal intoxication. Analysis comprised short hair segments and their initial wash water solutions. In the intoxications, morphine and hydromorphone levels were 3.3 to 56 pg/mg and at maximum 9.8 pg/mg, respectively. Both levels and hydromorphone to morphine ratios were significantly lower compared to chronic morphine use. In the non-fatal intoxication, the highest hydromorphone to morphine ratio was obtained in the segment corresponding to the time of intoxication. Morphine ratios of wash to hair were significantly higher in the intoxications compared to chronic use, being indicative of sweat/sebum contamination. We recommend including the analysis of hydromorphone and the initial wash solution in cases of morphine intoxications. Our study demonstrates that hydromorphone to morphine ratios can help in distinguishing single from chronic morphine use and in estimating the period of exposure when a consecutive hair sample can be collected in survived intoxications.

## 1. Introduction

### 1.1. Background

Morphine is an opioid drug indicated for the management of severe pain and the substitution maintenance treatment of opioid dependents. In cases of suspected morphine intoxications, forensic toxicologists are most commonly faced with the question of opioid tolerance to be answered in front of the legal authority. Tolerance is defined as a reduced responsiveness to an opioid drug leading to the need to use increasing doses to achieve the desired effect [1]. Hence, tolerance occurs upon chronic morphine use. The knowledge of the individual’s history of opioid use is therefore of paramount importance when interpreting blood or tissue concentrations. As there is no measure of tolerance in these types of samples, hair analysis remains the only tool to exclude or provide indication of tolerance. For this purpose, segmental testing of scalp hair should be performed as it provides time-resolved retrospective monitoring of drug use [2,3].

Based on the concept that drugs circulating in the bloodstream are incorporated into the growing hair within the hair follicle, a hair sample represents the individual’s drug consumption in the months prior to its collection depending on hair length [4]. Hair follicles are embedded in the epidermal epithelium approximately 3 to 4 mm below the skin’s surface [5]. Scalp hair grows at a rate of 0.6 to 1.4 cm per month [3]. Consequently, the time delay between drug incorporation into hair and drug appearance in hair reaching the skin surface ranges between 6 to 20 days, with an average of 13 days.

Although the precise mechanism is still not fully understood, further incorporation pathways of drugs into hair have to be considered in the interpretation of results [4]. First, there is evidence that external contamination from drug powder or smoke may lead to positive hair findings [3,6]. Second, several studies have demonstrated that drugs enter hair by self-contamination via sweat/sebum following use leading to drug detectability in hair segments which have grown before intake [7,8,9,10].

One common side effect of opioid use is sweating [11], which may have implications for opioid testing in hair and its interpretation. While one or two controlled medical applications of morphine in the scope of a surgery were reported to be only detectable in the segment corresponding to the period of treatment [12], reported fatal morphine intoxications in individuals not being known as drug addicts resulted in the detection of morphine in the whole tested hair length of 6 cm at concentration levels of 490 to 570 pg/mg hair [13] and 43 to 46 pg/mg hair [14]. In each intoxication case, the presence of morphine was explained by excessive sweating during the agony period. Kintz proposed that the presence of homogenous consecutive concentrations in segmental analysis may be considered indicative of potential contamination from the individual’s body fluids [14]. However, the indisputable distinction between morphine contamination by body fluids such as sweat/sebum and chronic morphine use remains ambiguous.

Notably, the consideration of metabolite to drug ratios in hair samples has been shown to help in the discrimination of external contamination by drug powder versus drug intake for, e.g., cocaine and tramadol [15,16]. With respect to morphine and heroin, the concentration ratios of morphine (Mor)’s minor metabolite hydromorphone (HyMor) in hair samples have been demonstrated to serve as discrimination tool [17].

Herein, we report on the investigation of metabolite ratios HyMor/Mor in segmental hair analysis to distinguish single from chronic morphine intake and to determine the time period of intake. The investigation was conducted on the basis of a non-fatal morphine intoxication case in which morphine tolerance and chronic morphine use were in question. The study comprises segmental hair testing in individuals following morphine intoxication and individuals with documented chronic morphine use. Additionally, analysis of hair wash solutions was performed to examine whether the concentration ratio of morphine in the initial wash solution to morphine in hair (wash/hair) can be indicative of contamination by sweat/sebum. In the intoxication cases, toxicological analysis also included blood and urine testing.

### 1.2. Case Series

Details on the morphine intoxication cases are summarized in Table 1.

#### 1.2.1. Case 1: Non-Fatal Morphine Intoxication

A 100-year old woman (51.2 kg, 175 cm) was found comatose, fade, and sweaty at a nursing home. Her respiratory rate was depressed to 4 to 8 breathings per minute. The nurse assumed a stroke and the woman was transferred to the hospital. At the emergency department, the urine screening was positive for opioids and benzodiazepines. As an overdose was assumed, multiple doses of naloxone and flumazenil were administered as antidotes. These administrations led to a significant improvement of her condition. According to the medical report, the woman has never been prescribed opioids. The attorney requested a comprehensive toxicological screening. Peripheral blood and urine samples were collected 4.5 h after finding her comatose at the nursing home. The woman survived the intoxication thanks to medical intervention. Subsequently, the question of opioid tolerance and single or chronic morphine administration was raised. Therefore, a 1st and 2nd scalp hair sample was collected 9 and 51 days after the incident, respectively. The woman had white hair without cosmetic treatment.

#### 1.2.2. Case 2: Fatal Morphine Intoxication

A 16-year-old girl was found dead after a party with suspected drug use. Femoral venous blood, urine, and hair samples were collected at autopsy one day after death to investigate the cause of death. The girl had dark brown hair without cosmetic treatment.

#### 1.2.3. Case 3: Fatal Morphine Intoxication

A 76-year-old woman was found dead at a nursing home. It was documented that 50 and 5 mg morphine were administered 4 and 1.5 h, respectively, before the time of death. She had never been prescribed opioids. It was questioned whether the morphine administration was correct. Femoral venous blood, urine, and hair samples were collected at autopsy five days after death. The woman had gray-dark brown hair without cosmetic treatment.

#### 1.2.4. Case 4: Fatal Morphine Intoxication

A 92-year old woman died at a nursing home. She had received an injection of 5 mg morphine 80 min and 3 h 20 min, respectively, according to a doctor’s prescription, before she had been found dead. As the cause of death was unknown and additional administration was suspected, a comprehensive toxicological screening was requested. Femoral venous blood, urine, and hair samples were collected at autopsy two days after death. The woman had white-gray hair without cosmetic treatment.

#### 1.2.5. Therapeutic, Chronic Morphine Use

Slow-release oral morphine (SROM) is an approved substitution maintenance treatment for opioid dependence [18]. Scalp hair samples of six different chronic morphine users from routine abstinence controls were considered for the study. All individuals administered SROM continuously at doses from 90 to 1200 mg daily.

## 2. Results

### 2.1. Morphine Intoxication Cases

All urine samples were positive for opioids in the cloned enzyme donor immunoassay (CEDIA) screening. The liquid chromatography-tandem mass spectrometry (LC-MS/MS) screening confirmed the presence of morphine in all samples. All blood samples were positive for morphine. Free morphine concentrations are given in Table 1. HyMor was not detected in any blood and urine sample.

**Figure 1:**
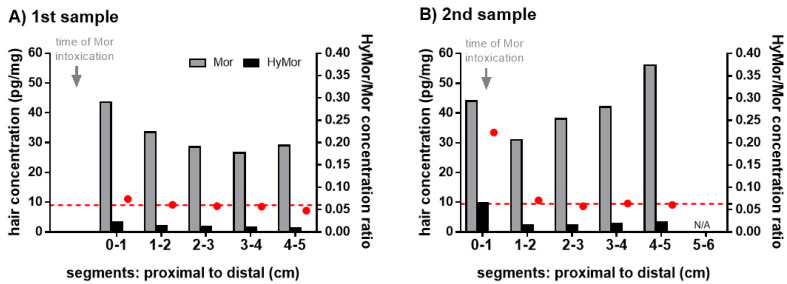
Morphine (Mor, gray bars) and hydromorphone (HyMor, black bars) concentrations (left y-axis) and HyMor to Mor concentration ratios (red circles, right y-axis) in the hair segments of the 100-year old victim’s 1st (**A**) and 2nd (**B**) sample collected 9 and 51 days after the Mor intoxication, respectively. The red dashed line represents the average of HyMor to Mor concentration ratios of the five segments in the 1st sample and four distal segments in the 2nd sample, respectively (HyMor/Mor in segment 0–1 of the 2nd sample: distinctly different).

The results of the segmental hair and hair wash solution analysis are summarized in Table 2 and Table 3. Morphine was detected in all tested segments in case 1, 2, and 3 at levels ranging from 3.3 to 56 pg/mg hair. Morphine concentrations decreased along the hair segments from proximal to distal in the 1st sample of the non-fatal intoxication case 1 (Figure 1), whereas they increased from proximal to distal in the fatal intoxication cases 2 and 3 (Table 2). HyMor was detected in all tested segments in case 1 and 2 at levels ranging from below LOQ to 9.8 pg/mg hair. HyMor/Mor ratios in hair ranged from 0.048 to 0.089 for all segments, except for the first segment of the 2nd sample (case 1) displaying an elevated value of 0.223 (Table 2). All tested hair segments of case 4 were negative for morphine and HyMor. In case 1, the comparison of the 1st and 2nd hair sample collected 9 and 51 days after morphine intoxication, respectively, showed no clear difference concerning morphine concentrations (Table 2). However, the HyMor concentration in the first segment of the 2nd hair sample corresponding to the time of morphine intoxication was substantially higher compared to all other segments of the 1st and 2nd hair sample in case 1 (Figure 1). The HyMor/Mor ratio in this segment was likewise distinctly higher compared to the other segments of the 1st and 2nd hair sample (Figure 1). The coefficient of variation (CV) of HyMor/Mor ratios in all segments of the 1st and 2nd sample was 66.6%; however, the CV decreased to 12.6% if the ratio of the 1st segment of the 2nd sample corresponding to the time of morphine intoxication was not considered.

The initial wash solutions of all hair segments of case 1 to 4 were positive for morphine, but negative for HyMor (Table 3). In the fatal case 4, morphine could be only found in the initial wash solutions, but not in the hair segments. The wash/hair ratio for morphine ranged from 0.16 to 0.76 in the non-fatal intoxication case 1 with sample collection 9 and 51 days following the incident, respectively, and from 1.1 to 3.5 in the post-mortem cases. In case 1, morphine concentrations in all wash solutions of the 1st hair sample were higher compared to those of the 2nd hair sample (Table 3).

### 2.2. Documented Chronic Morphine Use

Mean morphine and HyMor concentrations and HyMor/Mor ratios along the totally tested hair length of six chronic morphine users are given in Table 4.

**Figure 2:**
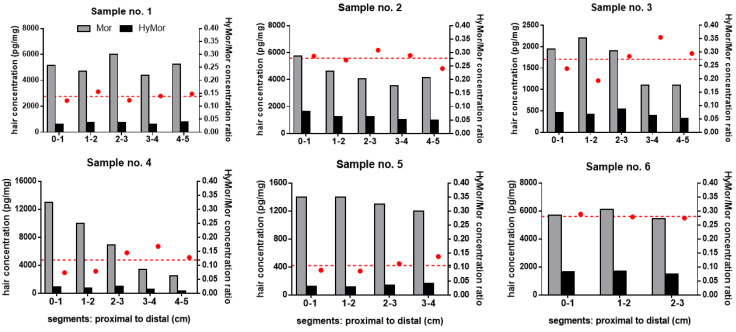
Morphine (Mor, grey bars) and hydromorphone (HyMor, black bars) concentrations (left y-axis) and HyMor to Mor concentration ratios (red dots; right y-axis) in the hair segments of six individuals (no. 1 to 6) with chronic Mor intake. The red dashed line represents the average of HyMor to Mor concentration ratios of the respective segments.

The correlation of daily morphine dose and the mean concentration along the hair segments was far stronger for HyMor compared to morphine (*p* < 0.001; Pearson coefficient R^2^ = 0.95 and 0.26, respectively). Morphine and HyMor concentrations and HyMor/Mor ratios per segment are displayed in Figure 2. Morphine and HyMor concentrations in hair segments ranged from 1100 to 13000 pg/mg and from 120 to 1700 pg/mg hair, respectively. Both levels were substantially higher compared to those in the intoxication cases. Morphine and HyMor concentrations were more or less uniform along the hair segments expect for sample no. 4.

HyMor/Mor ratios ranged from 0.074 to 0.36 (median: 0.17). They were likewise more or less consistent along the hair segments, with CVs ranging from 2.6% (sample 6) to 35% (sample 4).

All initial hair wash solutions were positive for morphine. Means are given in Table 4. Wash/hair ratios for morphine along all hair segments ranged from 0.0095 to 0.16 (median: 0.045), which was significantly lower compared to wash/hair ratios in the intoxication cases (Table 3 and Table 4). Hair wash solutions of 5 out of 6 individuals were positive of HyMor. Wash/hair ratios for HyMor ranged from 0.0035 to 0.13 (median: 0.015). The HyMor-negative wash solutions derived from hair segments of sample no. 5 which displayed the lowest morphine concentrations.

## 3. Discussion

In morphine intoxications, it is crucial to reveal whether the victim or decedent was opioid-tolerant or opioid-naïve. In cases with a lack of background information, the preferred specimen to be analyzed is hair, due to the ability to document time-resolved long-term drug exposure.

Herein, we present concentrations of morphine and its metabolite HyMor in hair segments and their initial wash water solutions in one non-fatal and three fatal morphine intoxication cases in which the individuals had never been prescribed opioids compared to those of individuals who administered morphine chronically as medication on a daily basis. In all intoxication cases, morphine intake was confirmed by the analysis of blood and urine samples. All blood and urine samples were negative for HyMor which may be a matter of method sensitivity or type of matrix analyzed.

In reported cases in the literature, morphine concentrations in hair segments of opioid-naïves following intake were consistent with those in our cases. Levels were 290 pg/mg in a 1.5-cm segment in a drug-facilitated crime in which morphine sedation was suspected to have occurred at least two times [19] and 11 pg/mg in a 2-cm segment in a single or double application in a clinical setting [12]. HyMor was not tested in these studies. It should be kept in mind that drug levels in hair always depend on the extraction method which was also demonstrated for morphine and HyMor [20].

In two fatal intoxications in our study (case 2 and 3), morphine concentrations increased along the hair segments from proximal to distal (Table 2), whereas they decreased in the non-fatal intoxication case 1 with 1st sample collection 9 days after the incident (Figure 1). The morphine concentration pattern along the segments in case 1 changed in the 2nd sample most probably because the hair proportion formed during the time of intoxication—represented by the first segment—has grown out (Figure 1). In the fatal intoxication case 4, all hair segments were negative for morphine and HyMor.

In the intoxication cases 1, 2, and 3, morphine concentrations were in the low range according to our own statistical data comprising all morphine-positive hair samples. They were significantly lower compared to those in chronic users and below 200 pg/mg, which is the cut-off stated by the Society of Hair Testing (SoHT) to identify use [6]. This underlines the importance of reporting drug concentrations not necessarily according to cut-off levels in forensic cases.

The chronic users’ morphine levels in all hair segments were far above the SoHT cut-off value of 200 pg/mg as expected. Interestingly, there was a strong positive relationship between daily dose and the mean concentration of HyMor but not morphine (Table 4). Morphine was taken as a slow-release oral dosage form. The slow release of morphine may have prevented the saturation of metabolizing enzymes responsible of the formation of HyMor, leading to a better correlation of the morphine daily dose to HyMor hair concentration. However, this correlation has to be investigated in further studies.

The distinction of single from chronic morphine intake based solely on morphine concentrations is not necessarily possible as the extent of incorporation is dependent on different factors such as hair color [3]. Additionally, drug levels in hair can be elevated in intoxication cases with excessive sweating during the agony [13], thereby mimicking repeated or chronic drug use. In fact, opioids are known to increase sweating. Sweat produced by eccrine glands, which are located in close association with the hair follicles, may contain drugs from passive diffusion from the blood stream or transdermal migration across the skin [21]. This is particularly the case for basic drugs as sweat is usually acidic and can serve as a trap [21]. To investigate this, the initial wash water solutions were analyzed and wash/hair ratios for morphine were calculated. Wash/hair ratios in the fatal intoxication cases were far above 0.5, which was stated to indicate that the source of drug in the wash solution is from external contamination [22] rather than incorporation from blood stream. The lower wash/hair ratios in the non-fatal intoxication case 1 can be explained by leaching of drug from hair by personal hygiene as the two hair samples were collected 9 and 51 days after the intoxication. Interestingly, in the fatal case 4, morphine could be only found in the wash solutions, but not in the hair segments. One possible explanation could be the relatively low dose of morphine which was administered just few hours before the time of death. In this case, it may have been too early for morphine incorporation into hair via sweat/sebum. Otherwise, morphine being only attached to the outer hair layer could have been completely removed by our in-house wash protocol. This was also demonstrated for, e.g., zolpidem [23]. Yet, this finding supports the importance of analyzing initial wash solutions in cases with negative hair sample results, particularly if hair is the only available specimen. Wash/hair ratios for morphine were by far lower in hair segments of chronic morphine users, indicating that the greatest morphine proportion is incorporated into the inner hair matrix by the blood stream, thereby being not easily removable by washing hair with water.

HyMor was detected in the intoxication case 1 and 2. HyMor/Mor ratios in all segments of these cases and those of the chronic morphine users were within the range as previously reported [17]. No striking HyMor/Mor ratio pattern along the hair segments of the fatal intoxication case 2 and of chronic morphine users were observed (Figure 2, Table 2). In contrast, in the non-fatal intoxication case 1 in which a 2nd hair sample could be collected 51 days after the incident, the proximal hair segment displayed a substantially higher HyMor/Mor ratio compared to the distal segments (Figure 1, Table 2). This segment corresponded to the time period of intoxication. Concomitantly, the HyMor concentration was highest in the corresponding proximal segment (Figure 1, Table 2). We concluded that the analytes were incorporated into this segment within the hair follicle directly from the blood stream. Morphine intake was proven in this period by the positive blood and urine testing. In case 1, HyMor/Mor ratios in the distal segments of the 2nd hair sample (Figure 1B) and in all segments of the 1st hair sample (Figure 1A) were in the same range. The 1st sample was collected 9 days after the incident. It is therefore rather unlikely that drugs incorporated in the hair follicle from the blood stream had already reached the skin surface, particularly considering that hair usually grows slower in older humans. The uniform and distinct HyMor/Mor ratios in these segments are, in our opinion, indicative of drug incorporation via sweat/sebum following only recent morphine use. Other authors reported on tilidine and tramadol concentrations in hair segments following a single dose [24,25]. Both opioid drugs were not only detected in the expected corresponding segments, but also in the distal ones. Moreover, tilidine was detected in hair already 24 h after a single intake [25]. Both findings suggest incorporation from sweat contamination as stated by the authors [24,25].

Our results indicate that the period of morphine use/administration could be estimated by investigating HyMor/Mor ratios along short hair segments in non-fatal cases in which a 2nd hair sample can be collected about 6 to 8 weeks after the incident. This may be especially helpful in cases with uniform morphine concentrations in segmental hair analysis which give no indication on the frequency or time of administration. It should be further investigated if HyMor/Mor ratios could also be helpful in the interpretation of survived heroin intoxications in which tolerance or repeated exposition in the past are in question.

Based on the presented cases and review of literature, we propose a scheme for the interpretation of analytical results in short hair segments tested in morphine intoxications, in order to get an indication of chronic/regular or only recent morphine use (Table 5).

## 4. Materials and Methods

### 4.1. Collection and Analysis of Blood and Urine Samples in the Intoxication Cases

Peripheral venous blood and urine samples in case 1 were collected by medical professionals at the hospital site. In the post-mortem cases 2, 3, and 4, femoral venous blood and urine samples were collected during the medico-legal autopsy. All samples were stored at –20 °C until analysis. Urine screenings were performed by CEDIA for common drugs on an Indiko Plus device and by an untargeted LC-MS screening method as described elsewhere [26]. The system consisted of a Thermo Fischer Ultimate 3000 UHPLC system (Thermo Fisher, San Jose, CA, USA) coupled to an ABSciex 3200 QTrap linear ion trap quadrupole mass spectrometer (ABSciex, Darmstadt/Germany). Automated MS matching was performed against a commercially available database [27]. Positive results were confirmed in blood by a subsequent confirmatory LC-MS/MS according to a previously validated method [28]. The system consisted of a Thermo Fischer Ultimate 3000 UHPLC system (Thermo Fisher, San Jose, CA, USA) coupled to an ABSciex 3200 QTrap linear ion trap quadrupole mass spectrometer (ABSciex, Darmstadt/Germany).

### 4.2. Hair Sample Collection and Analysis

All head hair samples were collected from the vertex posterior region by cutting a hair lock as close as possible to the scalp by trained professionals. During autopsy, hair samples were collected before opening the body to avoid contamination by body fluids. Samples were stored under dry conditions, at room temperature, and in the dark according to the guidelines of the SoHT [6].

**Figure 3:**
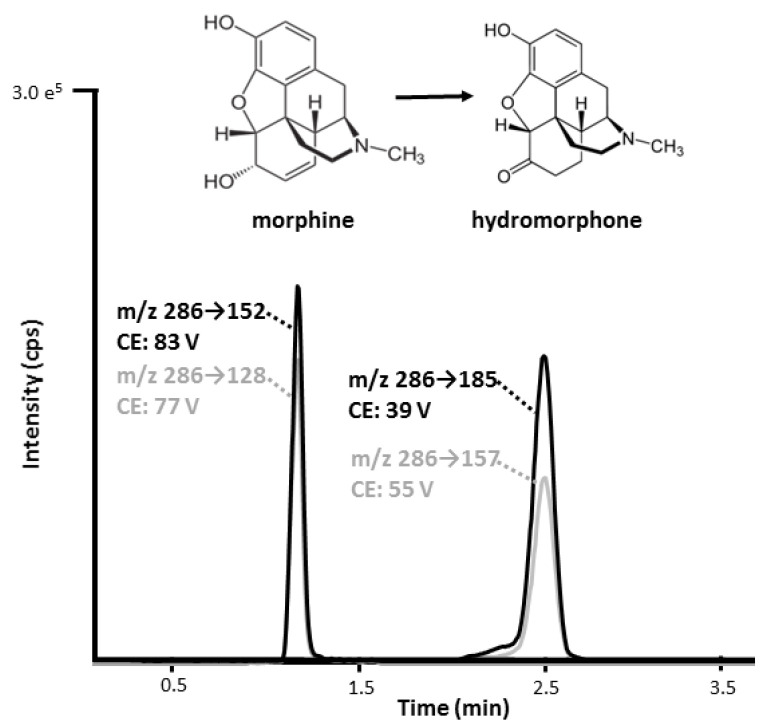
LC-MS/MS chromatogram of morphine and hydromorphone with their respective multiple reaction monitoring (MRM) transition and collision energy (CE). Quantifier and qualifier MRM transitions are marked in black and gray, respectively.

In the intoxication cases, hair samples were aligned and cut into three to five segments with a length of 1 to 3.5 cm (Table 2). Hair samples of individuals with documented chronic morphine use were aligned and segmented into three to five 1-cm segments depending on hair length. All hair samples were processed by a validated and routinely used method as previously published [29]. Hair decontamination was performed according to international guidelines [6]. In brief, hair was decontaminated successively with 15 mL water, 10 mL acetone, and 10 mL hexane. The first wash of 15 mL water was kept for further analysis. For each sample, on average 20 mg hair were weighed and pulverized. An internal standard solution consisting of deuterated analogues was added and analytes were extracted in a two-step extraction with, first, 1 mL methanol and, second, 1 mL methanol/2 mM ammonium formate buffer pH 3.5 (1/1, *v*/*v*), under shaking for 90 min in each extraction step. After each extraction step, samples were centrifuged, and the extraction solvent was removed for evaporation under nitrogen. The extracts were combined and dried residues were reconstituted in methanol/2 mM ammonium formate buffer pH 3.5, and analyzed by LC-MS/MS. The analytical method has been successfully applied in Proficiency Testing. The lower limits of quantification for morphine and HyMor were 1 and 2 pg/mg hair, respectively [29]. A representative LC-MS/MS chromatogram is illustrated in Figure 3. The initial wash water solutions were spiked with the above mentioned internal standard solution and acidified with 15 uL formic acid, evaporated under nitrogen at 40 °C overnight, and equally processed as hair samples.

## 5. Conclusions

The presented intoxication cases demonstrate the value of the metabolic ratio HyMor/Mor in the interpretation of segmental hair testing results. In non-fatal intoxication cases with sample collection shortly after the incident and about 6 to 8 weeks later, HyMor/Mor ratios may be indicative of the incorporation pathway (blood versus sweat), and hence, the differentiation of single from chronic morphine use (Table 5). Furthermore, they may help estimating the time of drug administration. In intoxication cases, we generally recommend the analysis of several short hair segments (1–2 cm length), metabolites, and the initial wash water. In our view, the following criteria should be considered in the interpretation of hair results: concentration level (low, medium, high) based on own statistical data collection, wash to hair and metabolite-to-parent-drug ratios, and their comparison along short hair segments (Table 5).

## Figures and Tables

**Table 1:** Details on morphine intoxication cases.

Case no.	Type	Sex	Age at Incident	Mor Dose	Hair Color	Total Tested Hair Length (cm)	Free Mor Conc. in Venous Blood (micro G/L)	Free HyMor Conc. in Venous Blood (micro G/L)	Urine LCMS/MS Screening *)
1	non-fatal	F	100	Unknown, most probably oral	White	1st and 2nd sample:5	180	nd	Mor + HyMor -
2	fatal	F	16	Unknown, most probably oral	Dark brown	8	740	nd	Mor + HyMor -
3	fatal	F	76	50 and 5 mg, subcutaneous	Gray-dark brown-	7	190	nd	Mor + HyMor -
4	fatal	F	92	5 mg twice, subcutaneous	White, few gray	10.5	260	nd	Mor + HyMor -

Mor: morphine; HyMor: hydromorphone; conc.: concentration; nd: not detected; *^)^ +: positive; -: negative.

**Table 2:** Results of hair segment testing in morphine intoxication cases *).

Hair Analysis	Mor Conc. (pg/mg)					HyMor Conc. (pg/mg)					HyMor/Mor Conc. Ratios					
Case 1 (1st sample: 1 + 1 + 1 + 1 + 1 cm) **)	44	34	29	27	29	3.3	2.1	1.7	1.5	1.4	0.075	0.062	0.059	0.056	0.048	
Case 1 (2nd sample: 1 + 1 + 1 + 1 + 1 cm) **)	44	31	38	42	56	9.8	2.2	2.2	2.7	3.4	0.223	0.071	0.058	0.064	0.061	
Case 2 (1 + 2 + 2 + 3 cm)	18	20	34	48	–	~1.6	~1.3	2.0	2.3	–	0.089	0.065	0.059	0.048		
Case 3 (2 + 2 + 3 cm)	3.3	3.6	4.8	–	–	nd	nd	nd	–	–						
Case 4 (2 + 2 + 3 + 3.5 cm)	nd	nd	nd	nd	–	nd	nd	nd	nd	–						

*) Segmental analysis from proximal to distal; Mor: morphine; HyMor: hydromorphone; conc.: concentration; **) 1st and 2nd hair sample was taken 9 and 51 days after the morphine intoxication, respectively; nd: not detected; –: remaining hair length was not considered for analysis; concentrations below the limit of quantification are marked by ~.

**Table 3:** Results of the initial hair wash solution testing in morphine intoxication cases *).

Analysis of Initial Hair Wash Solution	Mor Conc. (pg/mg)					HyMor Conc. (pg/mg)					Wash/Hair Conc. Ratios for Mor					
Case 1 (1st sample: 1 + 1 + 1 + 1 + 1 cm) **)	22	25	22	20	17	nd					0.50	0.74	0.76	0.74	0.59	
Case 1 (2nd sample: 1 + 1 + 1 + 1 + 1 cm) **)	6.9	6.5	8.4	9.7	11	nd					0.16	0.21	0.22	0.23	0.19	
Case 2 (1 + 2 + 2 + 3 cm)	48	60	120	120	–	nd	nd	nd	nd	–	2.7	3.0	3.5	2.5		
Case 3 (2 + 2 + 3 cm)	3.5	4.6	8.8	–	–	nd	nd	nd	–	–	1.1	1.3	1.8			
Case 4 (2 + 2 + 3 + 3.5 cm)	5.2	4.3	5.3	10	–	nd	nd	nd	nd	–						

*) Segmental analysis from proximal to distal; Mor: morphine; HyMor: hydromorphone; conc.: concentration; **) 1st and 2nd hair sample was taken 9 and 51 days after the morphine (Mor) intoxication, respectively; nd: not detected; –: remaining hair length was not considered for analysis; concentrations below the limit of quantification are marked by ~; wash/hair: concentration ratio of initial wash solution to hair.

**Table 4:** Details on chronic morphine users.

Sample No.	Sex	Age	Daily Mor Dose (mg)	Hair Color	Total Tested Hair Length (cm)	Mean Mor Conc. of 1-cm Hair Segments (pg/mg)	Mean HyMor Conc. of 1-cm Hair Segments (pg/mg)	Mean HyMor/Mor Ratio of 1-cm Hair Segments	Mean Mor Wash/Hair Ratio of All 1-cm Hair Segments
1	M	50	320	Dark brown-white	5	5283	702	0.14	0.012
2	M	56	400	Gray-white	5	4800	1383	0.28	0.11
3	M	52	120	Brown-gray	5	2017	477	0.27	0.052
4	M	54	400	Dark brown-gray	5	9967	917	0.12	0.033
5	M	59	90	Dark brown-gray	4	1325	130	0.11	0.028
6	M	52	1200	Brown-gray	3	5750	1617	0.28	0.10

Mor: morphine; conc.: concentration; HyMor: hydromorphone.

**Table 5:** Summary and recommendations for interpretation of short hair segment results tested in morphine-positive cases.

Hair Segment			Initial Hair Wash		Wash/Hair Ratio	Main Incorporation Pathway Indicating the Specified Mor Use
Mor (pg/mg) *)	HyMor	HyMor/Mor	Mor	HyMor	Mor	
>200	positive	>0.1	positive	positive	<0.2	via blood; regular, repeated/chronic Mor use
<200	positive	>0.1	positive	positive	<0.2	via blood; single/rare/occasional Mor use
>200	positive	<0.1	positive	positive	>0.2	via sweat/sebum and additionally via blood; potentially regular/chronic Mor use
<200	positive	<0.1	positive	positive	>0.2	via sweat/sebum; only very recent Mor use
positive	nd		positive	positive or nd	>0.2	via sweat/sebum; only very recent Mor use
nd	nd		positive	nd		via sweat/sebum; only very recent Mor use

*) Society of Hair Testing cut-off value for morphine use: 200 pg/mg hair; Mor: morphine; HyMor: hydromorphone; nd: not detected; wash/hair: concentration ratio of wash solution to hair.

## Data Availability

The datasets generated during and/or analyzed during the current study are available from the corresponding author on reasonable request.
